# Perioperative hypothermia and its clinical consequences in geriatric patients undergoing abdominal surgery: a retrospective cohort study

**DOI:** 10.1007/s11845-026-04387-y

**Published:** 2026-04-29

**Authors:** Meliha Orhon Ergun, Seniyye Ulgen Zengin

**Affiliations:** Department of Anesthesiology and Reanimation, Marmara University School of Medicine, Istanbul, Turkey

**Keywords:** Abdominal surgery, Anesthesia complications, Geriatric patients, Perioperative hypothermia, Postoperative outcome, Thermoregulation

## Abstract

**Objective:**

Geriatric patients are highly susceptible to perioperative hypothermia due to diminished physiological reserve, comorbidities, and polypharmacy. Even mild temperature reductions may contribute to cardiac instability, delayed emergence from anesthesia, coagulopathy, increased blood loss, and elevated infection risk. This study aimed to evaluate the clinical impact of perioperative hypothermia in geriatric patients undergoing abdominal surgery.

**Study design:**

Retrospective observational study.

**Methods:**

Medical records of 226 geriatric patients (ASA II–III) who underwent abdominal laparotomy between September 2018 and June 2022 at Marmara University were reviewed. Core temperature, anesthesia technique, operative duration, hemodynamic parameters, delayed emergence, postoperative intensive care unit (ICU) need, wound infection, mortality, and hospital length of stay were analyzed. Patients were classified as hypothermic (< 36.0 °C at any intraoperative measurement) or normothermic. Statistical analyses included Chi-square, Fisher’s exact, and Mann–Whitney *U* tests.

**Results:**

Perioperative hypothermia occurred in 195 patients (86.3%). Compared with normothermic patients, those who developed hypothermia had significantly higher rates of delayed emergence (*p* = 0.015), greater postoperative ICU requirement (*p* = 0.006), and longer hospitalization (*p* < 0.001). Hypothermic patients received higher doses of general anesthetic agents (*p* < 0.001). Heart rate was significantly lower at the 105th minute in the hypothermic group (*p* < 0.05), while instantaneous hypothermia was associated with higher baseline and 15-min heart rate and mean arterial pressure (*p* < 0.05). Other perioperative variables did not differ significantly.

**Conclusion:**

Perioperative hypothermia is highly prevalent among geriatric abdominal surgery patients and is associated with delayed emergence, increased ICU need, and prolonged hospitalization. These findings emphasize the need for routine temperature monitoring and active warming strategies to enhance perioperative safety in this vulnerable population.

## Introduction

Geriatric patients represent a uniquely vulnerable surgical population due to their diminished cardiopulmonary reserve, increased comorbidity burden, and frequent exposure to polypharmacy [1]. Among the challenges encountered in this demographic, perioperative hypothermia—defined as a core temperature below 36.0 °C—remains one of the most pervasive and clinically consequential complications. Prior evidence consistently demonstrates its association with higher perioperative morbidity and mortality, particularly in elderly individuals whose thermoregulatory capacity is intrinsically impaired [2, 3]. Notably, as much as 65% of hypothermic episodes occur within the first hour of surgery, despite the use of active warming strategies, underscoring the rapidity with which heat loss can occur under anesthetic conditions [4].

Heat dissipation during the perioperative period results from a combination of conduction, convection, radiation, and evaporation—mechanisms that disproportionately affect older adults owing to age-related reductions in vasoconstrictive responses, impaired shivering, and altered metabolic heat production [5]. Even mild deviations from normothermia have been linked to a spectrum of adverse outcomes, including increased cardiac events, coagulopathy, greater intraoperative blood loss, delayed anesthetic emergence, impaired wound healing, and a heightened risk of surgical site infection [3]. These complications collectively contribute to prolonged hospitalization and increased healthcare utilization.

Multiple physiological and patient-related factors further heighten susceptibility to perioperative temperature dysregulation in older adults. Advanced age, frailty, low baseline core temperature, and low body mass index are well-recognized contributors. Chronic systemic conditions such as diabetes mellitus, thyroid dysfunction, paraplegia, and polytrauma amplify this vulnerability by disrupting normal autonomic and metabolic responses [6]. Anesthetic agents compound these risks: benzodiazepines, opioids, propofol, and volatile anesthetics blunt the hypothalamic thermoregulatory threshold, whereas ketamine largely preserves sympathetic autonomic activity and therefore exerts minimal thermoregulatory impact [5, 7]. Although neuromuscular blocking agents do not directly alter temperature control, hypothermia markedly prolongs their duration of action—indeed, a mere 2 °C reduction in core temperature may double the duration of neuromuscular blockade [8].

Surgical factors also play a substantial role in intraoperative heat loss. Low ambient operating room temperatures, extensive skin exposure, large incisions, prolonged surgical times, cold insufflation gases, and unwarmed irrigation fluids synergistically promote hypothermia during abdominal procedures [5, 9]. Given the multifactorial etiology of perioperative temperature decline, continuous, accurate, and site-appropriate monitoring—using the distal esophagus, tympanic membrane, nasopharynx, or pulmonary artery—is essential for timely detection and intervention [3].

Considering the heightened susceptibility of geriatric patients and the broad array of complications associated with even mild hypothermia, a clear understanding of its physiological consequences and its effect on postoperative recovery is of substantial clinical importance. This study therefore aims to evaluate the impact of perioperative hypothermia on intraoperative physiological parameters and postoperative outcomes in geriatric patients undergoing abdominal surgery.

## Materials and methods

This retrospective, single-center observational cohort study was conducted following approval from the Clinical Research Ethics Committee of Marmara University Faculty of Medicine (Approval No: 09.2022.1462; November 4, 2022). Medical records of geriatric patients (≥ 65 years; ASA II–III) who underwent elective abdominal laparotomy between September 2018 and June 2022 at Marmara University Hospital were systematically reviewed.

### Inclusion and exclusion criteria

Eligible participants included patients aged ≥ 65 years who underwent elective abdominal laparotomy and had complete perioperative core temperature measurements. Exclusion criteria were established a priori to minimize heterogeneity and included:

- emergency surgical procedures;
- incomplete or missing perioperative temperature data;
- intraoperative use of extracorporeal circulation or deliberate therapeutic hypothermia;
- re-operations during the same hospitalization; and
- preoperative sepsis or hemodynamic instability.

These criteria ensured a clinically homogeneous cohort and reduced confounding related to severity of illness and procedural complexity.

### Data collection and definitions

Demographic characteristics, comorbidities, anesthesia technique, operative duration, intraoperative hemodynamic parameters, delayed emergence from anesthesia, postoperative intensive care unit (ICU) admission, wound infection, mortality, and length of hospital stay were extracted from electronic medical records.

In our institution, opioid-sparing or multimodal analgesic strategies may be used at the discretion of the attending anesthesiologist. However, during the study period there was no standardized institutional protocol for non-opioid anesthesia. Therefore, anesthetic management—including opioid administration—was individualized according to patient characteristics, surgical requirements, and the clinical judgment of the anesthesiologist. Because of the retrospective design of the study, opioid dosage variability could not be fully standardized across patients.

Core body temperature was measured at 15-min intervals using calibrated nasopharyngeal or tympanic thermometers of the same brand throughout the study period. When both modalities were recorded for a single patient, nasopharyngeal measurements were preferentially used to maintain methodological consistency. Perioperative hypothermia was defined as a core temperature < 36.0 °C at any intraoperative timepoint. Missing temperature values were managed through complete-case analysis.

Active warming devices such as forced-air warming systems and warming blankets are routinely available in all operating rooms in our institution. These devices are commonly used intraoperatively according to clinical judgment. However, due to the retrospective design of this study, the timing of warming initiation—particularly preoperative warming before induction—was not consistently documented in the medical records and therefore could not be analyzed systematically.

The ambient operating room temperature in our institution is generally maintained between 20 °C and 23 °C according to hospital environmental standards. Nevertheless, exact temperature recordings for individual procedures were not available in the retrospective dataset and therefore could not be incorporated into the statistical analysis.

Delayed emergence from anesthesia was defined as failure to achieve adequate spontaneous ventilation and purposeful responsiveness within 20 min after discontinuation of anesthetic agents. ICU admission followed institutional criteria, including hemodynamic instability, respiratory failure, and major postoperative complications.

### Bias control measures

To mitigate selection bias, all consecutive eligible patients during the study period were included without sampling. Measurement bias was limited by the standardized use of identical, hospital-wide calibrated temperature devices. No formal sample size calculation was performed given the retrospective nature of the study; however, the sample reflects a real-world, consecutive geriatric surgical population, thereby enhancing external validity.

### Statistical analysis

Categorical variables were summarized as frequencies and percentages, and continuous variables as medians with minimum–maximum ranges. Normality of distribution was assessed using the Kolmogorov–Smirnov test. As continuous variables exhibited non-normal distributions, between-group comparisons were performed with the Mann–Whitney *U* test. Categorical variables were compared using the Chi-square test or Fisher’s exact test when appropriate. Statistical significance was defined as a two-sided *p*-value < 0.05. All analyses were conducted using IBM SPSS Statistics for Windows (Version 20; IBM Corp., Armonk, NY, USA).

## Results

A total of 226 geriatric patients met the inclusion criteria and were included in the final analysis. Baseline demographic and clinical characteristics—including age, sex distribution, ASA physical status, and comorbidity profiles—were comparable between the hypothermia (*n* = 195) and normothermia (*n* = 31) groups, ensuring an appropriate basis for outcome comparison (Table 1).

**Table 1:** Baseline Demographic Characteristics of Hypothermic and Normothermic Patients

Variable	Hypothermia (*n* = 195)	Normothermia (*n* = 31)	*p*-value
BMI Category			0.532ᵃ
Normal	95 (48.7%)	12 (38.7%)	
Overweight	86 (44.1%)	17 (54.8%)	
Obese	14 (7.2%)	2 (6.5%)	
BMI (kg/m^2^)	25 (17–36)	25 (19–35)	0.225ᵇ

ᵃChi-square test

ᵇMann–Whitney *U* test

BMI, Body mass index

### Incidence of perioperative hypothermia

Across the nine scheduled intraoperative temperature measurements obtained at 15-min intervals, at least one episode of hypothermia (< 36.0 °C) was documented in 195 patients, corresponding to an overall incidence of 86.3%. **The median core temperature was significantly lower in the hypothermia group compared with the normothermia group** (*p* < 0.001), confirming substantial intraoperative temperature variability within this cohort (Table 2).

**Table 2:** Intraoperative Clinical Variables by Hypothermia Status

Variable	Hypothermia (*n* = 195)	Normothermia (*n* = 31)	*p*-value
Operative time (min)	120 (100–285)	120 (120–250)	0.056ᵇ
Duration of anesthesia (min)	150 (100–300)	150 (120–285)	0.708ᵇ
Anesthesia method			< 0.001ᵃ
— General anesthesia	148 (75.9%)	7 (22.6%)	
— Regional anesthesia	47 (24.1%)	24 (77.4%)	

ᵃChi-square test

ᵇMann–Whitney *U* test

### Postoperative recovery and clinical outcomes

Perioperative hypothermia was associated with markedly poorer postoperative recovery parameters.

Delayed emergence from anesthesia occurred with significantly greater frequency among hypothermic patients than among normothermic individuals (*p* = 0.015). Similarly, postoperative ICU admission was required in 17.4% (34/195) of patients in the hypothermia group, whereas only one patient in the normothermia group required ICU-level care (*p* = 0.006).

Length of hospital stay was also significantly prolonged in patients who developed hypothermia (Table 3; *p* < 0.001).

**Table 3:** Postoperative Complications by Hypothermia Status

Variable	Hypothermia (*n* = 195)	Normothermia (*n* = 31)	*p*-value
Delay in awakening	60 (30.8%)	3 (9.7%)	0.015ᵃ
ICU need	34 (17.4%)	0 (0.0%)	0.006ᶜ
Mortality	11 (5.6%)	0 (0.0%)	0.694ᶜ
Wound infection	22 (11.3%)	1 (3.2%)	0.216ᵃ
Hospital stay (days)	5 (4–9)	4 (4–6)	< 0.001ᵇ

ᵃChi-square test

ᵇMann–Whitney *U* test

ᶜFisher’s exact test

ICU: Intensive care unit

Mortality was observed in 11 patients (5.6%) in the hypothermia group, while no deaths occurred in the normothermia cohort. Although numerically different, this finding did not reach statistical significance (*p* = 0.694).

### Anesthetic requirements

Patients who experienced perioperative hypothermia required significantly higher doses of general anesthetic agents compared with those who maintained normothermia throughout the procedure (*p* < 0.001). This finding suggests a meaningful increase in anesthetic requirements in the presence of temperature dysregulation (Table 4).

**Table 4:** Intraoperative Heart Rate and MAP in Hypothermic vs Normothermic Patients

Time (min)	Hypothermia Median (Min–Max)	Normothermia Median (Min–Max)	*p*-valueᵃ
Heart Rate (bpm)			
0 min	89 (50–119)	75 (47–155)	0.024
15 min	76 (50–126)	71 (39–112)	0.022
30 min	67 (43–121)	65 (40–117)	0.296
45 min	67 (32–111)	64 (38–114)	0.256
60 min	66 (33–115)	66 (50–104)	0.812
75 min	65 (32–118)	67 (50–97)	0.376
90 min	65 (44–105)	68 (45–93)	0.139
105 min	64 (40–104)	68 (50–87)	0.011
120 min	65 (40–110)	66 (40–110)	0.799
MAP (mmHg)			
0 min	102 (81–149)	80 (30–152)	< 0.001
15 min	77 (48–145)	72 (38–117)	0.018
30 min	70 (30–107)	72 (30–121)	0.107
45 min	70 (35–106)	71 (44–137)	0.237
60 min	69 (42–107)	70 (42–120)	0.747
75 min	71 (38–147)	72 (43–117)	0.375
90 min	70 (38–122)	72 (41–116)	0.204
105 min	70 (38–116)	71 (38–113)	0.349
120 min	72 (38–117)	70 (50–136)	0.804

All measurements were obtained at 15-min intervals during the intraoperative period

^a^Mann–Whitney *U* test

MAP: Mean arterial pressure

### Hemodynamic parameters

When heart rate and MAP values were evaluated across the entire intraoperative timeline, the mean heart rate at the 105th minute was significantly lower in the hypothermia group compared with the normothermia group (*p* < 0.05), whereas MAP values remained broadly comparable between groups at most time points (Table 4).

Instantaneous hypothermia analysis demonstrated that heart rate was significantly higher at 0, 15, and 105 min in hypothermic patients (*p* = 0.024, *p* = 0.022, and *p* = 0.011, respectively). Similarly, MAP values were significantly higher at baseline and 15 min (*p* < 0.01 and *p* = 0.018). Hemodynamic parameters at all other time points did not differ significantly between the two groups (Table 5).

**Table 5:** Heart Rate and Mean Arterial Pressure at Instantaneous Hypothermia Timepoints

Time (min)	Hypothermia Median (Min–Max)	Normothermia Median (Min–Max)	*p*-valueᵃ
Heart Rate (bpm)			
0 min	76 (47–155)	70 (49–86)	0.056
15 min	72 (39–126)	66 (51–84)	0.186
30 min	66 (40–121)	68 (52–85)	0.644
45 min	66 (32–114)	68 (51–90)	0.870
60 min	65 (33–115)	70 (50–85)	0.469
75 min	65 (32–118)	68 (55–88)	0.191
90 min	65 (44–105)	69 (54–93)	0.091
105 min	64 (40–104)	70 (54–87)	0.009
120 min	65 (40–110)	70 (53–89)	0.105
MAP (mmHg)			
0 min	81 (30–152)	78 (53–116)	0.133
15 min	74 (38–145)	70 (49–96)	0.683
30 min	72 (30–121)	72 (40–107)	0.800
45 min	70 (35–137)	74 (44–99)	0.077
60 min	69 (42–120)	70 (42–100)	0.311
75 min	71 (38–147)	73 (45–100)	0.215
90 min	70 (38–122)	72 (41–106)	0.702
105 min	70 (38–116)	74 (38–103)	0.387
120 min	72 (38–136)	73 (50–110)	0.907

Instantaneous hypothermia” indicates the timepoint at which a temperature < 36.0 °C was recorded

^a^Mann–Whitney *U* test

MAP: Mean arterial pressure

### Postoperative complications

Wound infections occurred in 22 of 195 patients (11.3%) in the hypothermia group, whereas no surgical site infections were identified in the normothermia group. No significant between-group differences were observed regarding intraoperative bleeding, arrhythmias, or hypotension beyond the time-dependent hemodynamic variations already described (Table 3).

A limited number of patients received intraoperative blood transfusions; however, the frequency was low and did not differ significantly between the hypothermia and normothermia groups. Therefore, transfusion was not included as a primary determinant in the comparative analysis.

## Discussion

The present study demonstrates that perioperative hypothermia is exceedingly common among geriatric patients undergoing abdominal surgery, with an incidence exceeding 86%, and that its occurrence is associated with a broad spectrum of clinically meaningful adverse outcomes. These include delayed emergence from anesthesia, increased postoperative ICU utilization, prolonged hospitalization, and higher rates of postoperative complications. Collectively, these findings reinforce the well-established vulnerability of elderly patients to temperature instability due to age-related impairments in vasoconstrictive responses, reduced metabolic heat production, and diminished physiologic reserve. Although perioperative hypothermia has been extensively examined in mixed adult surgical populations, evidence focusing specifically on geriatric individuals—particularly studies incorporating multiple, high-frequency intraoperative temperature measurements—remains limited [10].

This study therefore provides valuable insight into the temporal dynamics and clinical consequences of intraoperative temperature decline in this high-risk population.

Hypothermia induces a series of predictable physiological alterations, most notably sympathetic nervous system activation with increased circulating catecholamines, which may produce tachycardia, hypertension, and increased myocardial oxygen demand [11, 12]. In frail geriatric patients with impaired cardiac reserve or underlying coronary artery disease, these hemodynamic perturbations may precipitate myocardial ischemia or arrhythmias [13]. In our cohort, instantaneous hypothermic episodes were characterized by early elevations in heart rate and MAP, consistent with an acute sympathetic surge. Interestingly, by the 105th minute, sustained hypothermia was associated with significantly lower heart rate values, suggesting a later phase of autonomic depletion—an observation that aligns with prior physiologic reports in elderly surgical patients. This biphasic pattern highlights the dynamic cardiovascular consequences of hypothermia and underscores the importance of vigilant intraoperative monitoring.

Hypothermia is also a potent disruptor of hemostasis, impairing platelet function and slowing coagulation enzyme activity [14]. Consequent coagulopathy may contribute to increased intraoperative blood loss and a higher likelihood of transfusion, which itself has been linked to immunomodulatory effects and greater postoperative infection risk [15]. Moreover, peripheral vasoconstriction reduces tissue oxygen tension, thereby creating a microenvironment conducive to bacterial proliferation [16, 17]. This mechanism likely contributes to the 11.3% wound infection rate observed in hypothermic patients in this study. Similar findings were reported by Yi et al., who demonstrated that hypothermia independently predicts postoperative complications and prolonged hospitalization [6].

Beyond cardiovascular and hematologic disturbances, hypothermia has notable implications for perioperative neurocognitive outcomes. Mild reductions in core temperature impair neuronal conduction, reduce cerebral metabolic rate, alter neurotransmitter balance, and disrupt autoregulatory mechanisms, thereby increasing susceptibility to postoperative delirium and cognitive dysfunction—conditions to which geriatric patients are already predisposed [18]. Previous research has linked impaired thermoregulation with both early postoperative delirium and longer-term cognitive decline [19]. Such complications substantially increase morbidity and length of stay and may prolong functional recovery, highlighting the broader systemic impact of perioperative temperature dysregulation.

Pharmacokinetic and pharmacodynamic alterations further complicate the anesthetic management of hypothermic patients. Reduced hepatic metabolism, decreased MAC requirements [11], and prolonged neuromuscular blockade—potentially doubling in duration with only a 2 °C temperature reduction [8]—may delay postoperative recovery. These physiologic mechanisms likely contributed to the significantly increased anesthetic drug requirements and delayed emergence from anesthesia observed in hypothermic patients in this study. Maintaining normothermia is therefore essential not only for physiologic stability but also for ensuring predictable anesthetic drug behavior and minimizing postoperative respiratory compromise or ICU admissions.

Although mortality differences between groups did not reach statistical significance, the occurrence of 11 deaths among hypothermic patients—compared with none in the normothermia group—aligns with prior observations linking thermoregulatory dysfunction and early postoperative mortality in elderly patients [20]. While causal inferences cannot be drawn due to the retrospective design, the observed numerical disparity underscores the potential lethality of perioperative hypothermia and strengthens the rationale for comprehensive prevention strategies.

Despite routine implementation of active warming measures—including forced-air warming and warmed intravenous fluids—more than 86% of patients in this study experienced at least one hypothermic episode. This high incidence suggests that current perioperative vigilance may be insufficient, and highlights the need for standardized institutional protocols that integrate consistent monitoring, early-warning triggers, and multimodal warming interventions [11, 21].

Differences in the timing and intensity of warming interventions, particularly the absence of routine prewarming protocols, may partially explain the high incidence of hypothermia observed in this cohort.

This study possesses several noteworthy strengths. Foremost is the relatively large sample of geriatric surgical patients—a demographic underrepresented in perioperative thermoregulation research. Additionally, the systematic acquisition of temperature measurements at fixed 15-min intervals provides a granular and physiologically informative profile of intraoperative thermal fluctuations, superior to the single-timepoint assessments used in many previous studies. The inclusion of detailed hemodynamic analyses further enriches the interpretation of hypothermia’s temporal cardiovascular impact.

Core temperature monitoring in our institution is most commonly performed using nasopharyngeal or tympanic thermometry. Although esophageal temperature probes are widely regarded as a reliable method for continuous core temperature monitoring during general anesthesia, they were not routinely used in the patient cohort analyzed in this retrospective study. Future prospective studies incorporating esophageal temperature monitoring may provide more precise assessment of intraoperative temperature dynamics.

In addition, ambient operating room temperature represents an important determinant of perioperative heat loss. Current perioperative guidelines recommend maintaining operating room temperatures of at least 21 °C to reduce the risk of intraoperative hypothermia, particularly in vulnerable populations such as geriatric patients.

However, certain limitations must be acknowledged. Because of the retrospective design and the relatively limited size of the normothermia group, multivariable regression analysis was not performed. Future prospective studies with larger balanced cohorts would allow more robust adjustment for potential confounders. The retrospective, single-center design may limit generalizability. Variability in anesthetic technique and warming strategies could not be fully controlled, although standard institutional practices were followed. Temperature measurements were obtained using both tympanic and nasopharyngeal methods, which may introduce measurement variability, despite consistent use of calibrated devices.

Tympanic measurements may be more susceptible to environmental influences compared with nasopharyngeal monitoring, which represents a limitation of retrospective datasets where monitoring techniques cannot be standardized.

Finally, unmeasured confounders—such as frailty, nutritional status, baseline cognitive function, or preoperative functional capacity—could have influenced postoperative outcomes.

The findings of this study underscore the urgent need for rigorous, protocol-driven perioperative temperature management in geriatric patients. Given the considerable morbidity associated with even transient hypothermic episodes—including cardiovascular instability, coagulopathy, wound complications, and neurocognitive impairment—hospitals should prioritize proactive prevention strategies. These include routine preoperative temperature screening, optimization of operating room environmental conditions, standardized use of forced-air warming devices, and administration of warmed intravenous fluids. Early identification and prompt correction of hypothermia may meaningfully reduce postoperative complications and enhance recovery trajectories in this vulnerable population. Future prospective multicenter studies incorporating standardized warming protocols and continuous core temperature monitoring are needed to better define optimal perioperative temperature-management strategies for geriatric surgical patients.

These findings highlight the importance of proactive perioperative temperature management in geriatric patients and suggest that even transient intraoperative hypothermic episodes may have clinically meaningful consequences.

## Conclusion

Perioperative hypothermia is highly prevalent among geriatric patients undergoing abdominal surgery and is associated with a range of clinically consequential adverse outcomes, including delayed anesthetic emergence, increased postoperative ICU admission, prolonged hospitalization, higher wound infection rates, and significant alterations in intraoperative hemodynamics. Given the impaired thermoregulatory capacity and limited physiologic reserve of elderly individuals, maintaining normothermia throughout the perioperative period is of critical clinical importance.

Immediate clinical strategies should focus on vigilant temperature monitoring and consistent use of active warming modalities. Long-term improvements will likely require adoption of standardized institutional temperature-management protocols supported by staff training and quality-improvement initiatives. Future prospective, multicenter studies are warranted to confirm these findings and to establish evidence-based multimodal warming strategies tailored to the geriatric surgical population.

## Data Availability

Available upon reasonable request.
